# Angiotensin type 1a receptor deficiency decreases amyloid β-protein generation and ameliorates brain amyloid pathology

**DOI:** 10.1038/srep12059

**Published:** 2015-07-08

**Authors:** Junjun Liu, Shuyu Liu, Yukino Matsumoto, Saki Murakami, Yusuke Sugakawa, Ayako Kami, Chiaki Tanabe, Tomoji Maeda, Makoto Michikawa, Hiroto Komano, Kun Zou

**Affiliations:** 1 Department of Neuroscience, School of Pharmacy, Iwate Medical University, 2-1-1 Nishitokuta, Yahaba, Iwate 028-3694, Japan; 2 Department of Biochemistry, Nagoya City University Medical School, 1-Kawasumi, Mizuho-cho, Mizuho-ku, Nagoya 467-8601, Japan

## Abstract

Alzheimer’s disease is characterized by neuronal loss and cerebral accumulation of amyloid-β protein (Aβ) and lowering the generation of Aβ is a pivotal approach in the strategy of Alzheimer’s disease treatment. Midlife hypertension is a major risk factor for the future onset of sporadic Alzheimer’s disease and the use of some antihypertensive drugs may decrease the incidence of Alzheimer’s disease. However, it is largely unknown how the blood pressure regulation system is associated with the pathogenesis of Alzheimer’s disease. Here we found that the deficiency of angiotensin type 1a receptor (AT1a), a key receptor for regulating blood pressure, significantly decreased Aβ generation and amyloid plaque formation in a mouse model of Alzheimer’s disease. The lack of AT1a inhibited the endocleavage of presenilin-1 (PS1), which is essential for γ-secretase complex formation and Aβ generation. Notably, the ligand of AT1a, angiotensin II, enhanced Aβ generation, PS1 endocleavage and γ-secretase complex formation. Our results suggest that AT1a activation is closely associated with Aβ generation and brain amyloid accumulation by regulating γ-secretase complex formation. Thus, removal of life style factors or stresses that stimulate AT1a to elevate blood pressure may decrease Aβ generation and brain amyloid accumulation, thereby preventing the pathogenesis of Alzheimer’s disease.

Alzheimer’s disease is the most common neurodegenerative disorder, defined by memory loss and increased presence of cerebral amyloid plaques^1^. Amyloid plaque is largely composed of neurotoxic amyloid-β protein (Aβ), which is generated from amyloid precursor protein (APP) via sequential cleavages by β- and γ-secretase^2^. Presenilin-1 (PS1) and -2 are the catalytic subunits of the γ-secretase. Missense mutations of PS and APP can constantly enhance Aβ production and cause familial Alzheimer’s disease in 5% of the patients^3^. However, in the remaining 95% cases of sporadic Alzheimer’s disease, an imbalance between Aβ production and clearance occurs in all patients defined as Alzheimer’s disease, which is caused by other unknown upstream events^4^.

Midlife hypertension is known as a major risk factor for the onset of Alzheimer’s disease and about 5% of Alzheimer’s disease cases are potentially attributable to midlife hypertension. In contrast, hypotension in late life was consistently associated with increased risk of Alzheimer’s disease and dementia, particularly in individuals who took antihypertensive drugs^5^,^6^,^7^. These findings suggest that the blood-pressure-regulating system may be closely involved in the pathogenesis of Alzheimer’s disease by an unknown mechanism. Angiotensin II (Ang II) is the key molecule in renin-angiotensin system to cause blood vessel constriction and stimulate the secretion of aldosterone, which increases the reabsorption of sodium and water to increase blood pressure. Angiotensin type 1a receptor (AT1a) is the pivotal receptor of Ang II for elevating blood pressure in response to dehydration, hemorrhage or environmental stresses^8^,^9^,^10^. We hypothesized that AT1a may be involved in the amyloid pathogenesis and Aβ production in Alzheimer’s disease. Here we report novel data indicating that AT1a deficiency significantly decreases brain amyloid deposition and Aβ production in an Alzheimer’s disease mouse model by regulating γ-secretase complex formation.

## Results

### *Agtr1a* deficiency reduces brain amyloid plaques

**Figure 1:**
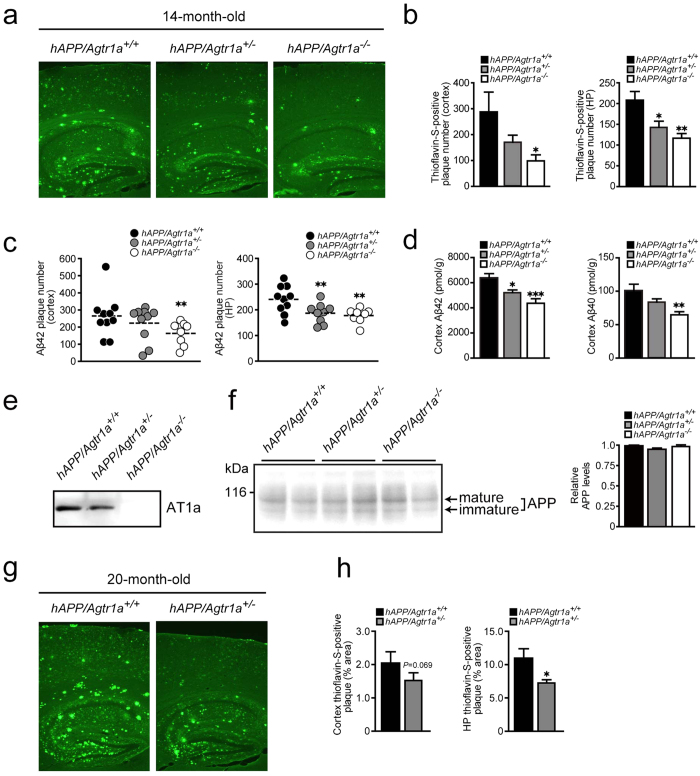
*Agtr1a* deficiency decreases Aβ deposition in an Alzheimer’s disease transgenic mouse model. (**a**) Comparison of amyloid deposition in the 14-month-old *hAPP/Agtr1a*^*+/+*^, *hAPP/Agtr1a*^*+/*−^ and *hAPP/Agtr1a*^−*/*−^ mouse brain by thioflavin-S staining. (**b**) Thioflavin-S-positive amyloid plaque number in the cortex and the hippocampus of 14-month-old mice. n = 4–8 *hAPP/Agtr1a*^*+/+*^ mice, n = 9 *hAPP/Agtr1a*^*+/*−^ mice and n = 6 *hAPP/Agtr1a*^−*/*−^ mice. (**c**) Quantification of Aβ42 plaque number in the cortex and hippocampus. n = 10 *hAPP/Agtr1a*^*+/+*^ mice, n = 9–10 *hAPP/Agtr1a*^*+/*−^ mice and n = 8 *hAPP/Agtr1a*^−*/*−^ mice. (**d**) Aβ42 and Aβ40 levels in the cortex of 14-month-old mice determined by ELISA. n = 5–9 *hAPP/Agtr1a*^*+/+*^ mice, n = 6–7 *hAPP/Agtr1a*^*+/*−^ mice and n = 5–6 *hAPP/Agtr1a*^−*/*−^ mice. (**e**) AT1a expression in *hAPP/Agtr1a*^*+/+*^, *hAPP/Agtr1a*^*+/*−^ and *hAPP/Agtr1a*^−*/*−^ cells was evaluated by immunoblot. (**f**) Expression of brain APP was evaluated by immunoblot analysis and densitometry. Mature and immature APP are indicated by arrows. (**g**) Thioflavin-S staining of brain sections from 20-month-old *hAPP/Agtr1a*^*+/+*^ and *hAPP/Agtr1a*^*+/*−^ mice. (**h**) Quantification of thioflavin-S-positive amyloid plaques in the cortex and hippocampus of 20-month-old *hAPP/Agtr1a*^*+/+*^ and *hAPP/Agtr1a*^*+/*−^ mice. n = 9 *hAPP/Agtr1a*^*+/+*^ mice, n = 9 *hAPP/Agtr1a*^*+/*−^ mice. Error bars show means ± s.e.m., *P < 0.05, **P < 0.01, ***P < 0.001 by one-way ANOVA followed by post hoc Bonferroni test comparing with *hAPP/Agtr1a*^*+/+*^ mouse. Cropped immunoblots are presented and all samples were compared under the same experimental conditions.

To evaluate the role of *Agtr1a* in amyloid plaque formation in the brain, we crossed *Agtr1a*-null mice and human APP (hAPP) transgenic mice bearing Swedish and Indiana mutations to generate *hAPP*/*Agtr1a*^+/−^ and *hAPP/Agtr1a*^−/−^ mice^11^,^12^. The body weights were comparable across genotypes and both the systolic and the diastolic blood pressure of the *hAPP/Agtr1a*^−/−^ mice were lower than the *hAPP/Agtr1a*^+/+^ or the *hAPP/Agtr1a*^+/−^ mice at age 14 months (Supplementary Fig. 1), consistent with previous findings in *Agtr1a*^−/−^ mice^11^. We first examined amyloid accumulation in the brain sections from 14-month-old mice using thioflavin-S, which binds to β-sheet-rich structures in amyloid fibrils and serves as an amyloid indicator. *hAPP/Agtr1a*^−/−^ mice showed significantly decreased amyloid plaques in the brain cortex and the hippocampus (Fig. 1a). Quantitative analysis revealed that the thioflavin-S-positive plaque number decreased 67% in the cortex and 43% in the hippocampus of *hAPP/Agtr1a*^−/−^ mice compared with *hAPP/Agtr1a*^+/+^ mice (Fig. 1b). In this Alzheimer’s disease mouse model, the longer and toxic form of Aβ, Aβ42, is the major component of the amyloid plaques, whereas a shorter and less toxic form of Aβ, Aβ40, comprises a very small portion of the amyloid plaques^13^. Immunostaining of Aβ42 using a specific anti-Aβ42 antibody also revealed significant decreases in the number of Aβ42 plaques in both the cortex and the hippocampus of *hAPP/Agtr1a*^−/−^ mice compared with *hAPP*/*Agtr1a*^+/+^ mice (Fig. 1c). Consistent with the thioflavin-S-staining and Aβ42-immunostaining results, the sandwich enzyme-linked immunosorbent assay (ELISA) showed that both Aβ42 and Aβ40 levels were decreased in the cortex of the 14-month-old *hAPP/Agtr1a*^−/−^ mice comparing with the age-matched *hAPP/Agtr1a*^+/+^ mice (Fig. 1d). The deletion of *Agtr1a* was confirmed in the primary cultured fibroblasts and the even expression of hAPP in the brain across genotypes was confirmed by Western blotting (Fig. 1e,f). To explore whether AT1a deficiency ameliorates amyloid pathology in the older Alzheimer’s disease mice, we examined the amyloid deposition in the 20-month-old mice. Because *hAPP/Agtr1a*^−/−^ mice had a higher mortality rate than *hAPP*/*Agtr1a*^+/+^ mice and most of them died before 20 months age, we were only able to compare *hAPP/Agtr1a*^+/+^ mice with *hAPP/Agtr1a*^+/−^ mice 20 months old. Similar to the results from 14-month-old mice, 20-month-old *hAPP*/*Agtr1a*^+/−^ mice also showed less thioflavin-S-positive amyloid plaques than *hAPP*/*Agtr1a*^+/+^ mice in both cortex and hippocampus (Fig. 1g,h).

### *Agtr1a* deficiency leads to decreased Aβ generation and γ-secretase complex formation

**Figure 2:**
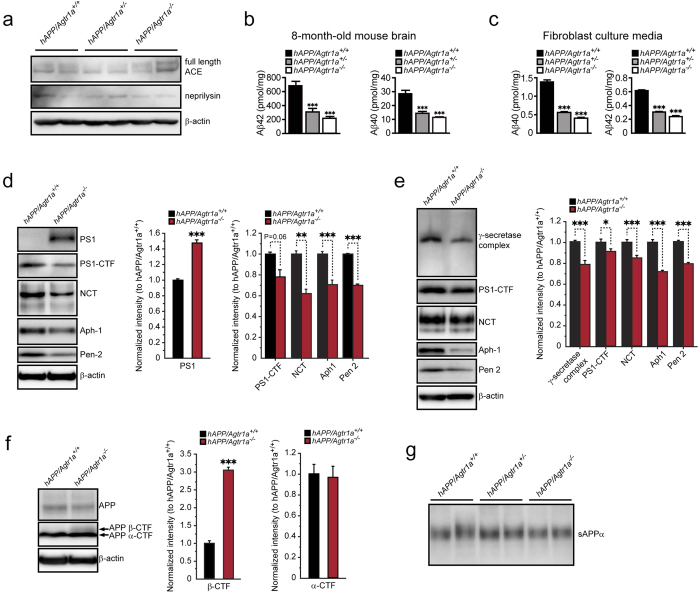
Aβ generation and γ-secretase components are decreased in *Agtr1a* deficient mouse brain and fibroblasts. (**a**) Effects of AT1a deficiency on ACE and neprilysin expression. (**b**) Determination of Aβ42 and Aβ40 levels in the brain cortex of 8-month-old *hAPP/Agtr1a*^*+/+*^, *hAPP/Agtr1a*^*+/*−^ and *hAPP/Agtr1a*^−*/*−^ mice by ELISA. n = 8 *hAPP/Agtr1a*^*+/+*^ mice, n = 9 *hAPP/Agtr1a*^*+/*−^ mice and n = 4 *hAPP/Agtr1a*^−*/*−^ mice. (**c**) Aβ40 and Aβ42 concentrations in the culture media of the primary cultured fibroblasts from *hAPP/Agtr1a*^*+/+*^, *hAPP/Agtr1a*^*+/*−^ and *hAPP/Agtr1a*^−*/*−^ mouse embryos. The Aβ40 and Aβ42 concentrations were normalized with the cellular protein amount. (**d**) Comparison of the γ-secretase components, PS1-CTF, NCT, Aph-1 and Pen-2, in the *hAPP/Agtr1a*^*+/+*^ and *hAPP/Agtr1a*^−*/*−^ mouse brain lysate by immunoblot analysis (left panels). The relative levels of the γ-secretase components were determined by densitometry with normalization to β-actin (right panels). (**e**) Comparison of the γ-secretase components in the cell lysate of *hAPP/Agtr1a*^*+/+*^ and *hAPP/Agtr1a*^−*/*−^ cells by immunoblot. (**f**) Amount of total cellular APP, β-CTF and α-CTF of APP (arrows) were determined by immunoblot analysis of APP. (**g**) sAPPα in the culture media of *hAPP/Agtr1a*^*+/+*^, *hAPP/Agtr1a*^*+/*−^ and *hAPP/Agtr1a*^−*/*−^ cells. Error bars show means ± s.e.m., n = 3–6 independent experiments. *P < 0.05, **P < 0.01, ***P < 0.001 by one-way ANOVA followed by post hoc Bonferroni test comparing with *hAPP/Agtr1a*^*+/+*^ mouse. Cropped immunoblots are presented and all samples were compared under the same experimental conditions. The whole panels of the immunoblots are displayed in Supplemental Fig. 4 for full length PS1 and PS1-CTF in the 14-month-old mouse brain (Supplemental Fig. 4a), and for γ-secretase complex in the cell lysate of *hAPP/Agtr1a*^*+/+*^ and *hAPP/Agtr1a*^−*/*−^ cells (Supplemental Fig. 4b).

The balance of Aβ generation and metabolism determines the extent of amyloid deposition in the brain. To investigate the mechanism by which AT1a deficiency leads to the decreased amyloid deposition, we first tested whether AT1a deficiency induces overexpression of angiotensin-converting enzyme (ACE), which converts angiotensin I to Ang II and may prevent Aβ deposition by converting and degrading Aβ42^14^. AT1a deficient mice did not show a compensated increase of ACE expression in the brain (Fig. 2a). Another Aβ-degrading enzyme, neprilysin, did not show any increase in the brain either (Fig. 2a). Because almost no amyloid deposits could be found in 8-month-old *hAPP* mice using thioflavin-S staining or Aβ immunostaining (data not shown), the brain Aβ levels determined by ELISA may reflect brain Aβ generation, but not Aβ deposition, in 8-month-old *hAPP* mice. By ELISA, we found that 8-month-old *hAPP/Agtr1a*^−/−^ and *hAPP/Agtr1a*^+/−^ mice had a significant lower Aβ42 and Aβ40 levels in the brain compared with *hAPP/Agtr1a*^+/+^ mice (Fig. 2b), suggesting decreased brain Aβ generation may occur in young *hAPP/Agtr1a*^−/−^ mice. To confirm this, we then measured Aβ levels in the culture medium of primary cultured mouse embryonic fibroblasts (MEFs) from *hAPP/Agtr1a*^+/+^, *hAPP/Agtr1a*^+/−^ or *hAPP/Agtr1a*^−/−^ littermates. As expected, the *hAPP/Agtr1a*^−/−^ cells showed significant decreases in both Aβ40 and Aβ42 levels, indicating that Aβ generation was impaired by *Agtr1a* deficiency (Fig. 2c). Aβ is generated from APP by β-secretase- and γ-secretase-mediated cleavage. α-secretase-mediated cleavage occurs between β-secretase- and γ-secretase-mediated cleavage, which prevents Aβ generation. The secreted APP by α-cleavage (sAPPα) showed a similar level in the culture media of *hAPP/Agtr1a*^+/+^, *hAPP/Agtr1a*^+/−^ and *hAPP/Agtr1a*^−/−^ cells, indicating that the α-secretase activity was not altered by *Agtr1a* deficiency (Fig. 2g). A γ-secretase complex is composed of presenilin-1 C- and N-terminal fragment, nicastrin (NCT), anterior pharynx-defective phenotype 1 (Aph-1) and presenilin enhancer 2 (Pen-2)^15^. The decreases in the levels of γ-secretase components, including PS1 C-terminal fragment (PS1-CTF), NCT, Aph-1 and Pen-2, were found in 14-month-old *hAPP*/*Agtr1a*^−/−^ mouse brain (Fig. 2d). Correspondingly, the levels of full length PS1 were increased in the *hAPP*/*Agtr1a*^−/−^ mouse brain (Fig. 2d, top panel). Quantitative analysis showed 12-39% decrease in PS1-CTF, NCT, Aph-1, Pen-2 and more than 1.4 times increase in full length PS1 in 14-month-old *hAPP*/*Agtr1a*^−/−^ mouse brain (Fig. 2d, right panel). Consistent with the results from mouse brain, *hAPP*/*Agtr1a*^−/−^ cells also showed decreased γ-secretase complex, PS1-CTF, NCT, Aph-1 and Pen-2 levels, indicating less assembly of γ-secretase complex (Fig. 2e). The quantitative analysis showed 8–30% decrease in γ-secretase complex, PS1-CTF, NCT, Aph-1 and Pen-2 levels in *hAPP*/*Agtr1a*^−/−^ cells (Fig. 2e, right panel). APP is cleaved by β-secretase to generate β-C terminal fragment of APP (β-CTF). The APP β-CTF is then cleaved by γ-secretase complex to generate Aβ. An accumulation of APP β-CTF was found in *hAPP*/*Agtr1a*^−/−^ cells, suggesting an attenuated γ-secretase activity (Fig. 2f). We further examined the messenger RNA (mRNA) levels of the four γ-secretase components. AT1a deficiency did not alter the mRNA levels of PS1, NCT, Aph-1 and Pen-2 in the primary cultured cells (Supplementary Fig. 2a–d). These results suggest that the decreased Aβ generation in *hAPP*/*Agtr1a*^−/−^ cells and mouse brain may be caused by the decreased formation of γ-secretase complex. Notch is also a substrate of γ-secretase^16^. However, AT1a deficiency did not show decreased intracellular domain of Notch (NICD) in the mouse brain or in the primary cultured cells, indicating the notch cleavage was not influenced by AT1a deficiency (Supplementary Fig. 3a and b).

### Ang II enhanced γ-secretase complex formation and Aβ generation through AT1a, PI3K and Akt pathway

**Figure 3:**
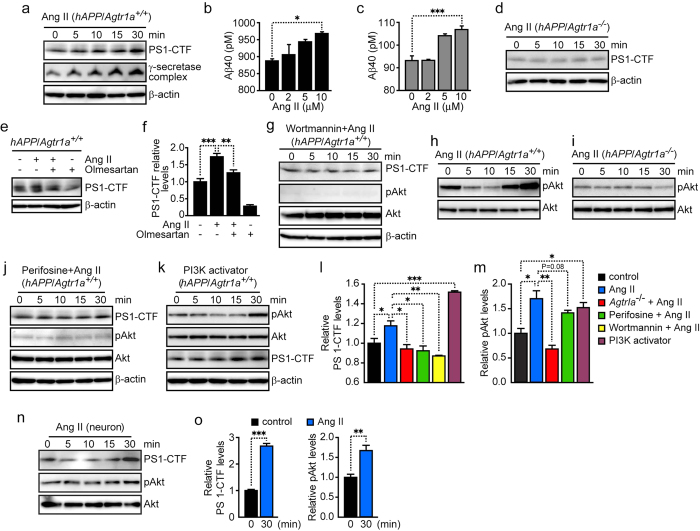
AT1a regulates PS1 endocleavage and the γ-secretase complex levels via PI3K-Akt pathway. (**a**) Immunoblot analysis of PS1-CTF and γ-secretase complex in *hAPP/Agtr1a*^*+/+*^ cells treated with Ang II. (**b**, **c**) Aβ40 and Aβ42 concentrations in the culture media of hAPP695 overexpressing fibroblasts treated with Ang II. (**d**) PS1-CTF levels in the *hAPP/Agtr1a*^−*/*−^ cells treated with Ang II were assessed by immunoblot analysis. (**e**, **f**) Olmesartan reversed the Ang II-stimulated increase of PS1-CTF levels and the relative levels of the PS1-CTF were determined by densitometry with normalization to β-actin. (**g**) Wortmannin blocked the Ang II-stimulated increase of PS1-CTF levels and the PI3K downstream phosphorylation of Akt in *hAPP/Agtr1a*^*+/+*^ cells. (**h**, **i**) The levels of phosphorylated and total Akt in *hAPP/Agtr1a*^*+/+*^ and *hAPP/Agtr1a*^−*/*−^ cells stimulated by Ang II. (**j**) Perifosine inhibited the increase of the PS1-CTF and the phosphorylated Akt induced by Ang II. (**k**) Phosphorylated Akt and PS1-CTF levels were increased by the treatment of a PI3K activator. (**l**) Comparative 30 minutes PS1-CTF levels of Fig. 3a,d,g,j,k. (**m**) Comparative 30 minutes pAkt levels of Fig. 3g–k. (**n**) Ang II treatment increased PS1-CTF and stimulated pAkt in primary cultured neuron. (**o**) The relative levels of the PS1-CTF and pAkt in primary cultured neuron after Ang II treatment 30 minutes were determined by densitometry with normalization to total Akt. Error bars show means ± s.e.m., n = 3–6 independent experiments. *P < 0.05, **P < 0.01, ***P < 0.001 by one-way ANOVA followed by post hoc Bonferroni test. Cropped immunoblots are presented and all samples were compared under the same experimental conditions. Full length immunoblots of PS1-CTF and γ-secretase complex in *hAPP/Agtr1a*^*+/+*^ cells treated with Ang II are available in Supplementary Figure 5a and b. Full length immunoblots of PS1-CTF in *hAPP/Agtr1a*^−*/*−^ cells treated with Ang II is available in Supplementary Figure 5c. Full length immunoblots of pAkt in *hAPP/Agtr1a*^*+/+*^ and *hAPP/Agtr1a*^−*/*−^ cells stimulated by Ang II are available in Supplementary Figure 5d and e.

AT1a is activated by its ligand Ang II, we therefore tested whether Ang II can promote PS1 endocleavage and γ-secretase complex formation. We found that Ang II treatment significantly induced increases in PS1-CTF levels and γ-secretase complex formation in *hAPP/Agtr1a*^+/+^ cells (Fig. 3a). Moreover, the treatment of Ang II enhanced Aβ40 and Aβ42 generation in human 695-amino acid amyloid precursor protein (hAPP695) overexpressing fibroblasts (Fig. 3b,c). In contrast to *hAPP/Agtr1a*^+/+^ cells, Ang II failed to stimulate the increase of PS1-CTF in *hAPP/Agtr1a*^−/−^ cells, suggesting that the decrease in PS1-CTF and γ-secretase complex levels in *hAPP/Agtr1a*^−/−^ cells may be caused by blocking of the Ang II signal (Fig. 3d). We further examined whether angiotensin receptor blocker (ARB), olmesartan, can inhibit the increase of PS1-CTF induced by Ang II. We found that olmesartan not only reversed the increase of PS1-CTF after Ang II treatment, but also decreased PS1-CTF levels in cells without Ang II treatment (Fig. 3e,f). Ang II can activate several signal transduction pathways through the protein kinase C, mitogen activated protein kinase, and phosphotidylinositide 3-kinases (PI3K) pathways^17^. Using specific inhibitors, we determined which pathway is involved in the Ang II-induced increase of PS1 endocleavage. A PI3K inhibitor, wortmannin, significantly inhibited the increase of PS1-CTF by Ang II, suggesting that Ang II promotes PS1 cleavage through activation of PI3K (Fig. 3g). Activated PI3K can phosphorylate its downstream signaling molecule, Akt. We found that Akt phosphorylation (pAkt) was stimulated by Ang II treatment in *hAPP/Agtr1a*^+/+^ cells, but not in *hAPP/Agtr1a*^−/−^ cells (Fig. 3h,i). As expected, perifosine, an Akt phosphorylation inhibitor, also inhibited the Ang II-induced increase of PS1-CTF (Fig. 3j). Both wortmannin and perifosine effectively inhibited Ang II-induced Akt phosphorylation (Fig. 3g,j). In addition to Ang II, a PI3K activator which activates Akt phosphorylation also increased PS1-CTF levels (Fig. 3k). The relative levels of PS1-CTF and pAkt at 30 min after Ang II treatment were compared (Fig. 3l,m). To confirm the effect of Ang II in the brain, we further examined the effect of Ang II on the primary cultured neurons. Ang II treatment significantly increased PS1-CTF and stimulated the pAkt (Fig. 3n,o). These results suggest that Ang II-AT1a-induced activation of the PI3K-Akt pathway can promote PS1 endocleavage and γ-secretase complex formation.

## Discussion

Pharmacological blockade of AT1a by ARBs is widely used to treat hypertension and administration of ARBs shows inverse associations with Alzheimer’s disease in hypertensive patients^18^, however, its mechanism remains largely unknown. Our results showed that AT1a deficiency significantly decreased amyloid deposition and Aβ generation by inhibiting γ-secretase complex formation and Aβ generation. Our study provides new insight into the mechanism of regulating γ-secretase complex formation and Aβ generation by the Ang II-AT1a pathway, suggesting that the elevation of Ang II and the activation of AT1a may increase Aβ generation and amyloid plaque accumulation. Reducing Aβ generation and deposition is targeted as therapies for the treatment of Alzheimer’s disease. AT1a is a kind of G protein-coupled receptors (GPCRs) and previous studies show that two GPCRs, G protein-coupled receptor 3 and β_2_-adrenergic receptor, mediate their effect on Aβ generation through interaction with β-arrestin 2 and Aph-1 subunit of the γ-secretase complex^19^,^20^. However, our results suggest that Ang II-AT1a signaling pathway, probably through PI3K and Akt, may promote PS1 endocleavage and γ-secretase complex formation, and then enhance Aβ generation. Thus, clinical trials using ARBs need to be performed for the treatment of Alzheimer’s disease in the patients without hypertension. In addition, the association between Ang II-AT1a activity and the risk of the onset of Alzheimer’s disease should be studied.

Aβ is generated from APP via sequential cleavages by β-secretase and γ-secretase complex. An active γ-secretase complex consists of endoproteolytic PS1, NCT, Aph-1 and Pen-2^2^. We found an increased β-CTF of APP (Fig. 2f) and a decreased PS1-CTF in the *hAPP*/*Agtr1a*^−/−^ mouse brain and cells (Fig. 2d,e), suggesting that AT1a deficiency decreases the endoproteolytic cleavage of PS1. Because the endocleavage of PS1 is required for the γ-secretase complex formation and activity, as expected, we further found that the levels of γ-secretase complex, PS1-CTF, NCT, Aph-1 and Pen-2 also decreased in AT1a deficient cells (Fig. 2e). However, quantitative real-time PCR data showed that the mRNA levels of PS1, NCT, Aph-1 and Pen-2 were not altered in the AT1a deficient cells, suggesting that AT1a deficiency did not change the expression of these γ-secretase components (Supplementary Fig. 2a–d). Therefore, it is possible that AT1a regulates the translation processes or the degradation of the γ-secretase components, or affects the reconstitution process of γ-secretase complex. As a GPCR, AT1a may also have the similar function with δ-opioid receptor that modulates the intracellular trafficking of the receptor/secretase complex to regulate APP processing^21^. The full length PS1 undergoes endoproteolysis to produce both an amino terminal and a carboxy terminal fragment, and is maintained at low steady level^22^. The full length PS1 was accumulated in 14-month-old *hAPP/Agtr1a*^−/−^ mouse brain (Fig. 2d and Supplementary Fig. 4a), but this accumulation was not found in *hAPP/Agtr1a*^−/−^ cells (Supplementary Fig. 5c). These findings suggest that the accumulation of the full length PS1 induced by AT1a deficiency is age-dependent or tissue-dependent.

Our results showed that β-CTF of APP increased in the *hAPP/Agtr1a*^−/−^ cells, whereas α-CTF of APP and NICD did not changed though all of them are the substrates of γ-secretase (Fig. 2f and Supplementary Fig. 3a and b). A previous study showed that β-CTF of APP is generated inside lipid rafts, whereas α-CTF is generated outside lipid rafts^23^. Interestingly, the γ-secretase complex was shown to be raft-associated^24^. Thus, α-CTF outside lipid rafts may not be readily cleaved by γ-secretase as β-CTF and the similar mechanism may also occur in the cleavage of Notch by γ-secretase. Alternatively, the γ-secretase may selectively cleave Notch with higher priority than β-CTF because Notch signaling is a vital factor for cell survival. Our results suggest that inhibition of AT1a may specifically reduce Aβ generation without impairing the cleavage of Notch.

Extensive studies suggest that decreasing amyloid β-protein generation and ameliorating brain amyloid pathology may have roles in preventing Alzheimer’s disease progression although stopping only one molecular pathway may not be enough to lead to a significant decrease of amyloid accumulation and clinical improvement^25^. Aβ42 is considered to have neurotoxic effects and Aβ40 has neuroprotective effects^26^,^27^,^28^,^29^. Thus, specifically inhibiting toxic Aβ42 generation appears to be a therapeutic strategy for Alzheimer’s disease treatment. In the Alzheimer’s disease mouse model we used, more than 90% amyloid deposition is Aβ42, whereas Aβ40 comprises a very small portion^13^. Our results suggest that inhibition of AT1a may partially prevent Aβ42 generation and deposition.

Compared with men, women are more susceptible to Alzheimer’s disease and this is considered as a consequence of estrogen deprivation after menopause. However, randomized controlled trials of hormone therapy in women did not show any protective effects against age-related cognitive impairment^30^. In normal women, Ang II is elevated during the luteal phase of the menstrual cycle^31^. Our results therefore imply a link between the AT1a activation and the susceptibility to Alzheimer’s disease in women.

Ang II and AT1a play the central role in renin-angiotensin system for maintaining blood pressure in response to dehydration, hemorrhage or environmental stresses. In most cases, the elevation of Ang II and the activation of AT1a are physiological responses and necessary. Nonetheless, our studies suggest that life style factors and environmental stresses that increase the blood pressure may also increase the risk of Alzheimer’s disease through Ang II-AT1a pathway.

## Materials and Methods

### Transgenic mice

We crossbred J20 mouse expressed hAPP bearing the Swedish and Indiana mutations under the control of the human platelet-derived growth factor beta polypeptide (PDGFB) promoter (The Jackson Laboratory) with *Agtr1a* deficient mice (The Jackson Laboratory) to generate heterozygous *hAPP/Agtr1a*^*+/*−^ and *Agtr1a*^*+/*−^ mice on the same C57BL/6 background. Then, we cross-mated *hAPP/Agtr1a*^*+/*−^ and *Agtr1a*^*+/*−^ mice to generate *hAPP/Agtr1a*^−*/*−^ mice. Mouse genotypes were determined by PCR on extract from tail cutting using the REDExtract-N-Amp Tissue PCR Kit (SIGMA). The age-matched heterozygous hAPP tagged *Agtr1a*^*+/+*^, *Agtr1a*^*+/*−^ and *Agtr1a*^−*/*−^ offspring were analyzed. All mice were bred on a 12-hour light/dark schedule with ad libitum access to food and water. All animal procedures were conducted in accordance with the Iwate Medical University Committee for Animal Use and all experimental protocols were approved by Iwate Medical University Committee.

### Immunohistochemistry

Mice were killed by carbon dioxide asphyxiation, then flush-perfused transcardially with phosphate buffered saline (PBS) containing 5 U/ml heparin (SIGMA). One hemibrain was fixed for 48 hours in 4% paraformaldehyde at 4 °C and equilibrated in 30% sucrose dissolved in PBS for 48 hours at 4 °C before being cut into 30 μm sections sagittally with a freezing microtome (Leica Microsystems). The other hemibrain was dissected into the hippocampus, cortex, thalamus, and brainstem as described previously to prepare for brain ELISA^14^. Thioflavin-S staining was applied as described previously to analyze the amyloid deposition in the mouse brain^13^. Immunostaining of the Aβ42 was performed by Vectastain ABC Kit (Vector Laboratories) using an anti-Aβ42 antibody (1 μg/ml) and a biotinylated goat anti-rabbit antibody (diluted at 1:1000, IBL) to determine the Aβ42 deposition in the mouse brain sections. Brain sections were imaged by a fluorescence microscope (BZ-9000, Keyence, Osaka, Japan). For 14-month-old mouse thioflavin-S staining and Aβ42 immunostaining, the number of Aβ plaques from 3–4 brain sections for each mouse was counted. For 20-month-old mouse thioflavin-S staining, the brain sections were analyzed by Metamorph Image Analysis software (Molecular Devices) to determine the area of the Aβ plaques. Aβ staining, imaging and analyzing were performed by investigators blinded to genotype.

### Primary culture

MEFs were prepared from E13.5 embryos as described previously^32^. Cells were cultured in 10% fetal bovine serum-DMEM (Wako). Experiments with primary cells were performed with the same passage time. Cerebral cortical neuronal cultures were prepared from Sprague Dawley rats at embryonic day 18 as described previously^27^.

### Aβ ELISA

Mouse cortices for ELISA were homogenized in 10 volumes of lysis buffer which contains 5.0 M guanidine·HCl/50 mM Tris·Cl, pH 8.0 (w/v), as described previously^33^. The brain homogenates were further diluted at 1:20 for 14-month-old Aβ40 ELISA and 8-month-old Aβ40 and Aβ42 ELISA, and at 1:2000 for 14-month-old Aβ42 ELISA in a dilution buffer provided with the ELISA kit (Wako, Osaka, Japan)^14^. Aβ40 and Aβ42 concentrations in the culture media were determined from 2–3 lines for each genotype of *hAPP/Agtr1a*^*+/+*^, *hAPP/Agtr1a*^*+/*−^ and *hAPP/Agtr1a*^−*/*−^ cells. *Agtr1a*^*+/+*^ primary cultured cells were infected with hAPP695 by retrovirus-mediated method according to published methods^34^. The infected hAPP cells were treated with Ang II to determine the effect of Ang II on Aβ generation. Aβ40 and Aβ42 concentrations were normalized to protein amount. All samples were measured in triplicate.

### Ang II treatment and cell extracts preparation

*hAPP/Agtr1a*^*+/+*^, *hAPP/Agtr1a*^*+/*−^ and *hAPP/Agtr1a*^−*/*−^ cells were grown up to 70% confluence and starved overnight in serum-free medium prior to treatment. Starved fibroblasts were administered 100 nM Ang II (Peptide Institute) for 5, 10, 15 and 30 minutes and washed with 1 mM sodium orthovanadate before being lysed with 20 mM HEPES pH 7.0, 0.5% deoxycholic acid, 0.15 M NaCl, 0.1% SDS, 1% Nonidet P-40, 4 mM EDTA, 10 mM NaF, 10 mM Na_4_P_2_O_7_, 2 mM sodium orthovanadate, containing a protease inhibitor cocktail (Roche). Olmesartan (1 μM, TRC), wortmannin (500 nM, Cell Signaling), perifosine (5 μM, Selleckchem) or PI3K activator (1 μg/ml, Santa Cruz Biotechnology) were added to the cells 2 h before Ang II treatment. The PI3K activator is a 1732.8 Da peptide with the sequence KKHTDDGYMPMSPGVA. This peptide binds to the SH2 domain of the PI3Kinase by the tyrosine phosphorylated version to activate the enzyme^35^.

### Blue native PAGE

Cells were suspended in a native sample buffer (Invitrogen) containing 1% digitonin and a protease inhibitor mixture. After centrifugation at 20,000 *g*, 4 °C for 30 minutes, the supernatant was separated onto the 4–16% Bis-Tris gel (Invitrogen) according to their molecular weight under the instructions of the Novex Bis-Tris gel system (Invitrogen). The γ-secretase complex levels were detected with a PS1-CTF antibody (Millipore).

### Immunoblotting

Equal amounts of protein from brain or cell lysate were separated by SDS-PAGE in 5–20% gel or blue native PAGE and blotted onto polyvinylidene difluoride (PVDF) membranes (Immobilon). The membranes were incubated with the primary antibodies overnight at 4 °C. Appropriate peroxidase-conjugated secondary antibodies were applied and the membranes were visualized by SuperSignal Chemiluminesence (Thermo Scientific). Membranes were stripped and reprobed with anti-β-actin antibody to normalize the loading amounts. Total Akt was detected on the same membrane after stripping the anti-pAkt antibody. The rabbit anti-Akt and anti-pAkt (Ser-473) antibodies were purchased from Cell Signaling. The anti-APP and anti-sAPPα monoclonal antibody, 22C11, was from Chemicon. The anti-AT1a antibody was purchased from Santa Cruz. The anti-nicastrin antibody, anti-C-terminus of APP antibody (A8717) and anti-β-actin antibody were purchased from Sigma-Aldrich. PS1-CTF antibody was from Millipore. The anti-Pen-2 antibody and anti-neprilysin antibody were purchased from Abcam. The anti-Aph-1 antibody was from COVANCE.

### Quantitative real-time PCR

The mRNA levels of PS1, NCT, Aph-1 and Pen-2 in *hAPP/Agtr1a*^+/+^ and *hAPP/Agtr1a*^−/−^ cells were compared by quantitative real-time PCR^36^. Briefly, RNA was extracted by ISOGEN (Nippon Gene) following the instructions of the manufacture. Extracted RNA was reverse-transcribed with ImProm-II reverse transcriptase (Promega). Real-time PCR was carried out by using FastStart Universal SYGR Green Master (Roche Applied Science) and specific oligonucleotide primer pairs for mouse PS1 (QT00098868, Qiagen), NCT (QT00198338, Qiagen), Aph-1 (QT00173894, Qiagen) and Pen-2 (QT00297927, Qiagen) according to the instructions of the manufacturer. Amplification and detection were performed using a 7500 fast real-time PCR system (Applied Biosystems) under the following conditions: 1 cycle each at 50 °C for 2 min and 95 °C for 10 min, 40 cycles each at 15 s and 60 °C for 1 min. Two lines of cells from each genotype of *hAPP/Agtr1a*^*+/+*^ and *hAPP/Agtr1a*^−*/*−^ mice were analyzed. All data were normalized to that of β-actin.

### Statistical analyses

For amyloid plaque deposition statistics, mouse brain sections were coded to ensure objective assessment, and codes were not broken until the analysis was complete. We compared group difference by one-way ANOVA followed by post hoc Bonferroni test for two or more groups against a control group. Two-tailed Student’s *t*-test was used to determine whether the results were significantly different between two groups. Statistical analysis was carried out using GraphPad Prism 5. *P*-value < 0.05 was considered to represent a significant difference. Graphs are expressed as means ± s.e.m.

## Additional Information

**How to cite this article**: Liu, J. *et al.* Angiotensin type 1a receptor deficiency decreases amyloid β-protein generation and ameliorates brain amyloid pathology. *Sci. Rep.* **5**, 12059; doi: 10.1038/srep12059 (2015).

## Supplementary Material

Supplementary Information
