# Supplementary material for: Angiotensin type 1a receptor deficiency decreases amyloid β-protein generation and ameliorates brain amyloid pathology
Source: Sci Rep. 2015 Jul 8;5:12059. doi: 10.1038/srep12059 (PMC4495558; doi:10.1038/srep12059)

Supplemantary Figures for

# Angiotensin type 1a receptor deficiency decreases amyloid β-protein generation and ameliorates brain amyloid pathology

Junjun Liu,[1] Shuyu Liu,[1] Yukino Matsumoto,[1] Saki Murakami,[1] Yusuke Sugakawa,[1] Ayako Kami,[1] Chiaki Tanabe,[1] Tomoji Maeda,[1] Makoto Michikawa,[2] Hiroto Komano,[1] Kun Zou[1]

[1]Department of Neuroscience, School of Pharmacy, Iwate Medical University, 2-1-1 Nishitokuta, Yahaba, Iwate 028-3694, Japan

[2]Department of Biochemistry, Nagoya City University Medical School, 1-Kawasumi, Mizuho-cho, Mizuho-ku, Nagoya 467-8601, Japan

Address correspondence to: Department of Neuroscience, School of Pharmacy, Iwate Medical University, Kun Zou or Hiroto Komano; Tel: 81-19-698-1820; Fax: 81-19-698-1864. Email: kunzou@iwate-med.ac.jp or hkomano@iwate-med.ac.jp

Figure legends

**Supplementary Figure 1.** AT1a deficient mice showed decreased systolic and diastolic blood pressure. The body weight (a), systolic and diastolic blood pressure (b and c) of the 14-month-old *hAPP/Agtr1a*$^{+/+}$, *hAPP/Agtr1a*$^{+/-}$ and *hAPP/Agtr1a* $^{-/-}$ mouse. n=8 *hAPP/Agtr1a*$^{+/+}$ mice, n=8 *hAPP/Agtr1a*$^{+/-}$ mice and n=8 *hAPP/Agtr1a*$^{-/-}$ mice. *$P < 0.05$, ***$P < 0.001$ by one-way ANOVA followed by post hoc Bonferroni test comparing with *hAPP/Agtr1a*$^{+/+}$ mouse.

**Supplementary Figure 2.** Real-time PCR quantitative analysis of PS1, NCT, Aph-1 and Pen-2 in AT1a deficient cells. The mRNA levels comparison of the four components of γ-secretase complex, PS1 (a), NCT (b), Aph-1 (c) and Pen-2 (d) between *hAPP/Agtr1a*$^{+/+}$ and *hAPP/Agtr1a* $^{-/-}$ cells. Error bars show means ± s.e.m., n=4 independent experiments, two-tailed Student's *t*-test.

**Supplementary Figure 3.** Notch cleavage did not change in AT1a deficient mouse brain and cells. The NICD levels were detected by Westernblot in the 14-month-old *hAPP/Agtr1a*$^{+/+}$, *hAPP/Agtr1a*$^{+/-}$ and *hAPP/Agtr1a* $^{-/-}$ mouse brain lysate (a), and in the *hAPP/Agtr1a*$^{+/+}$ and *hAPP/Agtr1a* $^{-/-}$ cells (b). Cropped immunoblots are presented and all samples were compared under the same experimental conditions.

**Supplementary Figure 4.** Original immunoblot panels corresponding to Fig. 2. The dashed boxes represent the bands shown in the original figure.

**Supplementary Figure 5.** Original immunoblot panels corresponding to Fig. 3.

Supplementary Figure 1
Liu J. et al.

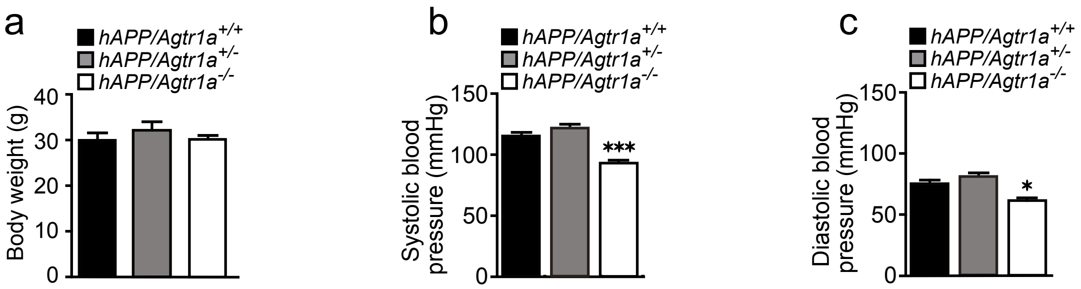

Supplementary Figure 2
Liu J. et al.

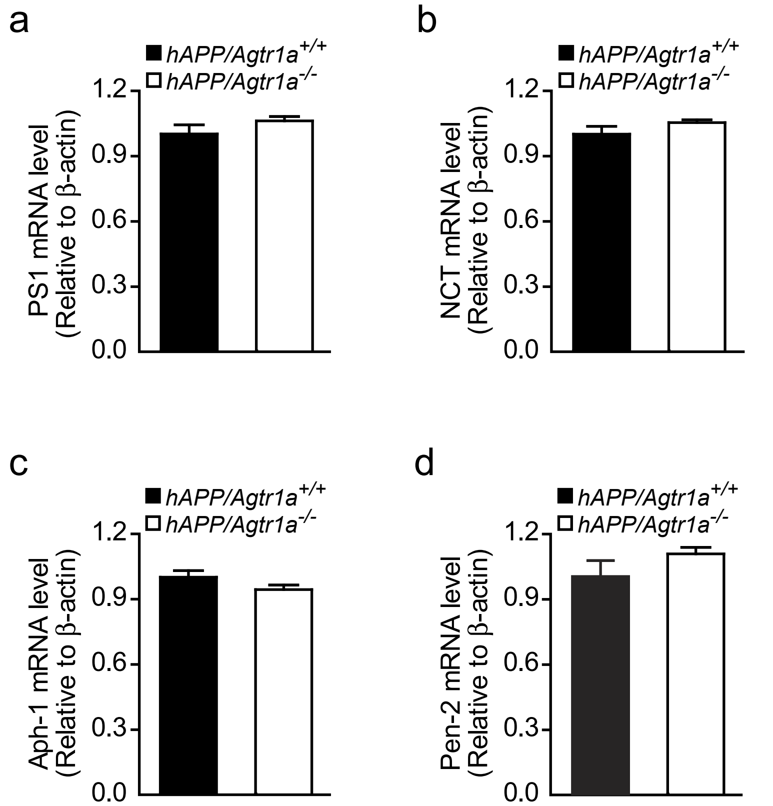

Supplementary Figure 3
Liu J. et al.

a

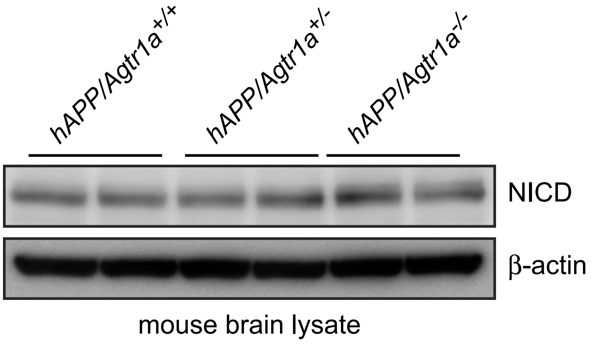


b

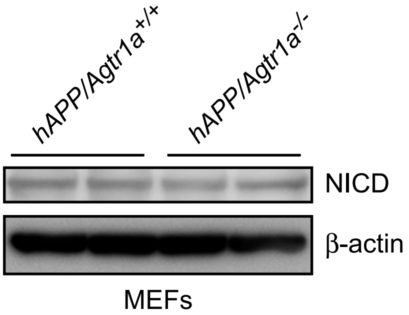

Supplementary Figure 4
Liu J. et al.

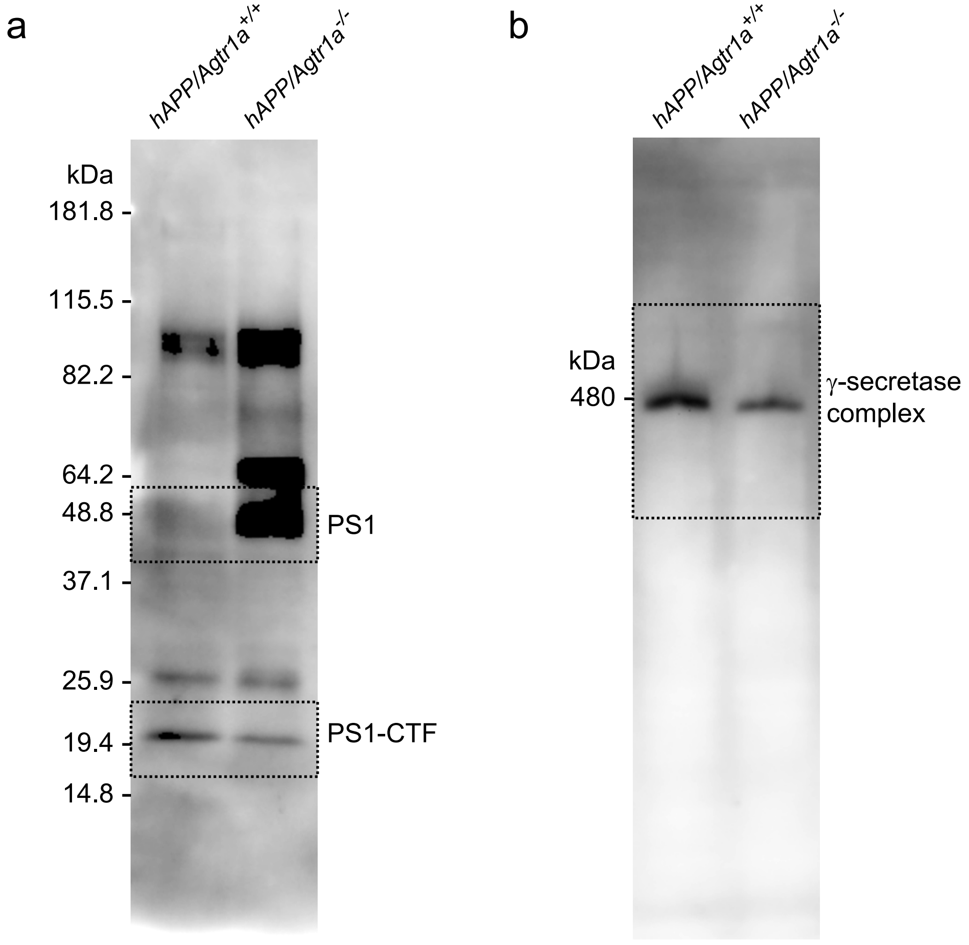

# Supplementary Figure 5
Liu J. et al.

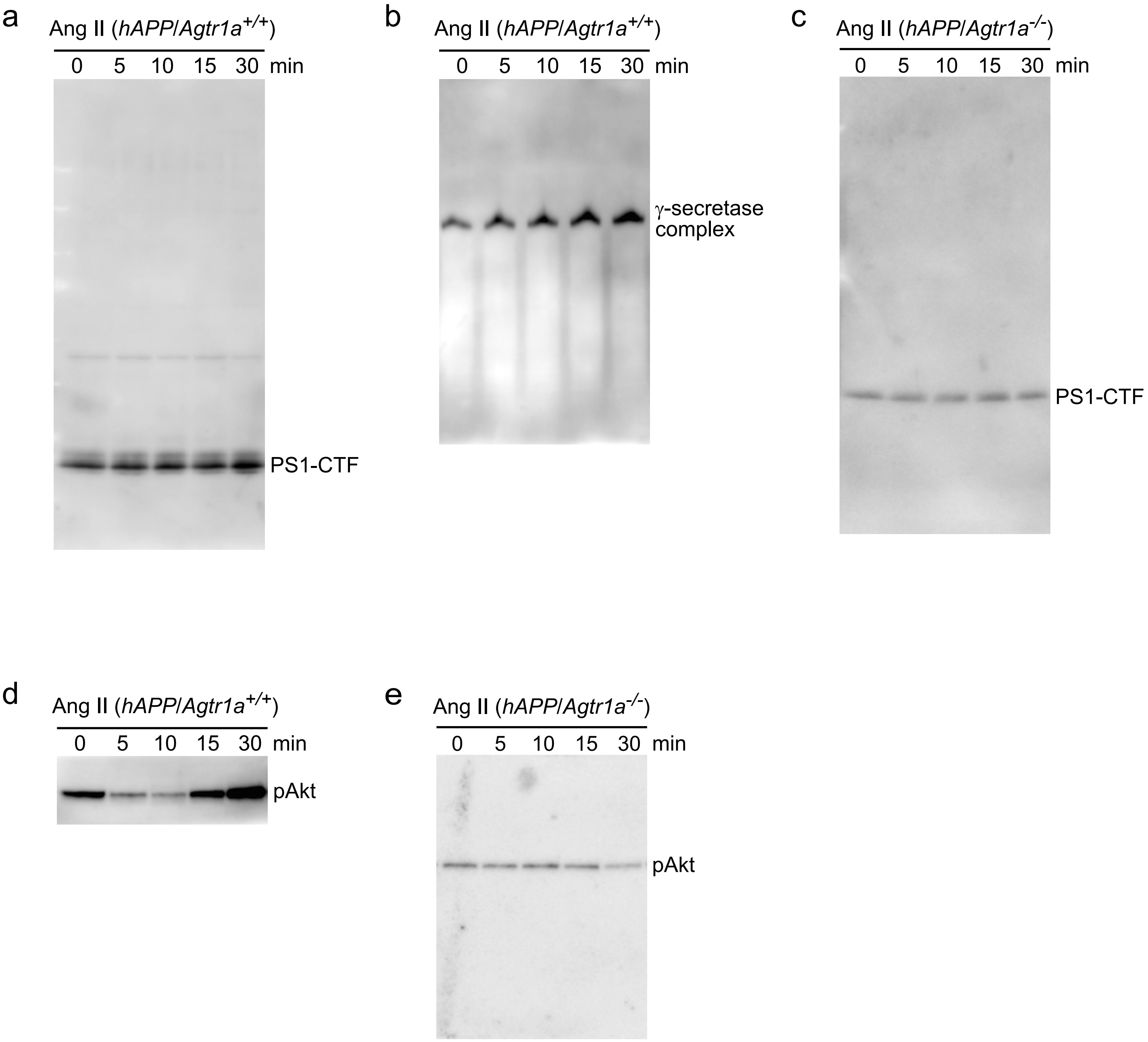

Supplement: Supplementary Information [file srep12059-s1.pdf]
